# Establishment of an Efficient In Vitro Propagation of *Cnidium officinale* Makino and Selection of Superior Clones through Flow Cytometric Assessment of DNA Content

**DOI:** 10.3390/genes13101815

**Published:** 2022-10-08

**Authors:** Hyung-Eun Kim, Jong-Eun Han, Hyoshin Lee, Hosakatte Niranjana Murthy, Hyuk-Joon Kwon, Gun-Myung Lee, So-Young Park

**Affiliations:** 1 Department of Horticultural Science, Chungbuk National University, Cheongju 28644, Korea; 2 Korea Disease Control and Prevention Agency, Osong-eup, Cheongju 28159, Korea; 3 Department of Forest Genetic Resources, National Institute of Forest Science, 39 Onjeong-ro, Suwon 16631, Korea; 4 Department of Botany, Karnatak University, Dharwad 580003, India; 5 Food Science R&D Center, Kolmar BNH Co., Seocho-gu, Seoul 30003, Korea

**Keywords:** flow cytometry, medicinal plant, multiple shoots, plant regeneration, rhizomes

## Abstract

*Cnidium officinale* is a valuable medicinal plant cultivated in Asia for its rhizomes. This study reports the in vitro regeneration of *Cnidium officinale* plants and the induction of rhizomes from microshoots. The rhizomatous buds of *Cnidium officinale* induced multiple shoots on Murashige and Skoog (MS) medium supplemented with 0.5 mg L^−1^ BA, which led to the regeneration of plants within four weeks of culture. After four weeks of culture, the plants were assessed for fresh weight, the number of leaves, the number of roots, and the length of roots to compare the performance of the different clones. The clones with good growth characteristics were selected with the aid of a flow cytometric analysis of 2C nuclear DNA content. The plants bearing high DNA values showed better growth characteristics. Various factors, namely, sucrose concentration (30, 50, 70, and 90 g L^−1^), ABA (0, 0.5, 1.0, and 2.0 mg L^−1^), the synergistic effects of BA (1.0 mg L^−1^) + NAA (0.5 mg L^−1^) and BA (1.0 mg L^−1^) + NAA (0.5 mg L^−1^) + ABA (1.0 mg L^−1^) with or without activated charcoal (1 g L^−1^), and light and dark incubation were tested on rhizome formation from microshoots. The results of the above experiments suggest that MS medium supplemented with 50 g L^−1^ sucrose, 1.0 mg L^−1^ ABA, and 1 g L^−1^ AC is good for the induction of rhizomes from the shoots of *Cnidium officinale*. Plantlets with rhizomes were successfully transferred to pots, and they showed 100% survival.

## 1. Introduction

*Cnidium officinale* Makino is an important herbaceous medicinal plant that belongs to the Apiaceae family. It is widely cultivated in China, Japan, and Korea for its rhizomes. Dried rhizomes are used in Chinese, Japanese, and Korean medicine as a tonic to improve blood circulation, overcome the problems of inflammation, and treat women’s menstrual problems [1]. The rhizomes of *Cnidium officinale* have been reported to possess several bioactive compounds, including polysaccharides [2,3,4] and volatile alkylphthalide derivatives [5,6]. Rhizome extracts and isolated compounds have been reported to exhibit various pharmacological activities, including antiangiogenic [7], anticancer [8,9,10], antidiabetic [11], anti-inflammatory [12,13], immunomodulatory [2], analgesic [14], and antimicrobial [15,16] effects. Yang et al. [17] demonstrated that the ethanolic rhizome extracts of *Cnidium officinale* are usable in the treatment of ischemic injury and, thus, that rhizomes could be used as recovery medicine after plastic surgery, for the disturbance of peripheral blood circulation, and for overcoming postmenopausal problems. Kim [18] and Cha [19] showed the positive effects of *Cnidium officinale* extracts on the repression of melanogenesis and in decreasing hyperpigmentation, as well as its use as a cosmetic ingredient.

*Cnidium officinale* does not set seeds; therefore, the multiplication of this plant only occurs through the division of rhizomes, and there is a high risk of virus dissemination affecting yield and quality during cultivation [1]. Therefore, in vitro propagation methods have been developed for this species using shoot tip culture and the induction of somatic embryogenesis using inflorescence and flowers [1,20,21]. However, plant regeneration efficacy is low and inconsistent. Further, *Cnidium officinale* is a rhizomatous plant, and the in vitro induction of rhizomes from regenerated shoots is desirable. In vitro rhizome induction has been reported in many bulbous plants, such as *Kaempferia galanga*, *Kaempfria rotunda* [22], *Curcuma longa* [23], and *Bambosa bambos* var. *gigantea* [24]. The in vitro production of rhizomes has commercial potential for the large-scale generation of pathogen-free seed material for propagation and utilization by the pharmaceutical industry. Further, in vitro rhizome production is also useful for storage, transport, and germplasm preservation. Therefore, in the present study, an efficient plant regeneration protocol was developed in *Cnidium officinale* using rhizome bud explants. The regenerated plants/clones were assessed for their DNA content using the flow cytometric method. Additionally, the DNA content of the regenerated clones was correlated with growth characteristics. In addition, a reliable method for in vitro rhizome induction from regenerated shoots was established.

## 2. Materials and Methods

### 2.1. Preparation of Plant Material and Induction of Microshoots from Rhizome Buds

The rhizomes of *Cnidium officinale* Makino growing in the greenhouse of Chungbuk National University, Korea, were used in the experiment. For the establishment of the in vitro culture of *Cnidium officinale*, buds (~1.5 cm long) collected from the rhizome were washed in running tap water for 1 h, and the bacteria and fungi were primarily removed by immersing them in a sterilizer containing 1000 ppm Physan 20 (Maril, Tustin, CA, USA) for 20 s. Subsequently, surface sterilization was conducted for 20 s with 70% ethanol and for 10 min in a solution with added Tween 20, surfactant (ThermoFisher, Seoul, Korea), and 2% NaOCl. The explants were thoroughly washed five to six times with sterile double-distilled water, and they were cultured on Murashige and Skoog [25] medium with 0.5 mg L^−1^ benzyl adenine (BA), 30 g L^−1^ sucrose, and 2.4 g L^−1^ Gelrite (Duchefa Biochimie, Haarlem, the Netherlands). The cultures were incubated at 25 ± 1 °C under a 16 h photoperiod under the white fluorescent light of 35 µ m^−2^ s^−1^. Subculture was conducted at four week intervals. The cultured explants developed shoots, simultaneously developing roots at the base of the shoots. Plantlets were maintained in MS medium with 0.5 mg L^−1^ BA through repeated subculturing. We compared the regeneration rate of ten different clones, which were available in our germplasm collection, and calculated the shoot proliferation rate in subsequent subcultures for up to three cycles.

### 2.2. Flow Cytometry

Flow cytometry was performed to determine the amount of 2C DNA in the in vitro-grown *Cnidium officinale* plants. Briefly, a fresh staining solution was prepared on the day of analysis by mixing the staining buffer with PI solution and RNase solution. Leaf discs (0.5 cm^2^) were prepared from the leaves of *Cnidium officinale* and *Nicotiana tabacum* cv. xanthi (internal standard) plants and placed in a Petri dish containing 200 µL of extraction buffer. Nuclei were isolated from the leaf discs using a CyStain PI Absolute P Kit (Partec, Görlitz, Germany), according to the manufacturer’s instructions. The solution was filtered through a 50 µm nylon mesh. Then, 800 µL of staining solution was added to the filtered solution in order to label the DNA with a fluorescent dye. The samples were incubated in the staining solution on a stationary surface in the dark for 20 min and then analyzed using CytoFLEX (Beckman Coulter Inc., Fullerton, CA, USA). At least 5000 nuclei were analyzed per sample.

### 2.3. Assessment of in Vitro Growth of Regenerated Plants

Ten plants regenerated in vitro from each clone of *Cnidium officinale* were selected, and they were re-cultured on regeneration medium (MS + 0.5 mg L^−1^ benzyl adenine, 30 g L^−1^ sucrose, and 2.4 g L^−1^ Gelrite) and maintained four weeks. After four weeks of culture, the plants were assessed for their plant height, their fresh weight, the number of leaves, and the number and length of roots. Subsequently, the plants were re-cultured for another passage of a four-week cycle, and the number of plantlets in each cycle and the shoot proliferation rate (shoot proliferation rate = the total number of shoots after subculture vs. the total number of shoots before subculture) were estimated.

### 2.4. In Vitro Induction of Rhizome

The microshoots (2 cm in length) obtained after the four passages of re-culturing were harvested and transferred to MS media supplemented with 30, 50, 70, and 90 g L^−1^ sucrose, or 0, 0.5, 1.0, and 2.0 mg L^−1^ abscisic acid (ABA) and 50 g L^−1^ sucrose for rhizome induction. Similarly, the effect of 0.5 mg L^−1^ naphthalene acetic acid (NAA) in combination with 1.0 mg L^−1^ BA alone, or in combination with 1.0 mg L^−1^ ABA with or without 1 g L^−1^ activated charcoal (AC) was also tested on the rhizome induction. In these treatments, the medium was supplied with 50 g L^−1^ sucrose. Depending on the experiment, the cultures were either kept in continuous dark conditions or in 12 h light (35 µmol m^−2^ s^−1^) and 8 h dark conditions at 25 ± 1 °C temperature. The cultures were maintained for up to eight weeks, and data were collected.

### 2.5. Acclimatization

One day before acclimatization, the lid of the culture vessel was opened by ~20% for adaptation to the ex vitro conditions. Then, the plants with rhizomes regenerated in vitro were harvested from the vessel, carefully washed with distilled water, and planted in wet Growfoam (Horticubes, Smithers-Oasis, Kent, OH, USA). The plants in Growfoam were placed in a plastic container (25 × 25 × 3 cm), and the water was changed every week. The plants were fertilized with 1.0 g L^−1^ Hyponex solution once per month. The transplanted plants showed 100% survival. After 3 months of growing ex vitro, the plants were transplanted and cultivated in plastic pots (diameter 10 cm) filled with an artificial soil mixture (peat moss 1:1 perlite and vermiculite 1). The potted plants were fertilized with 1.0 g·L^−1^ Hyponex solution (N20:P20:K20) once per month.

### 2.6. Statistical Analysis

A one-way analysis of variance (ANOVA) was performed to determine significant differences between the results of shoot/plant regeneration, flow cytometric data, the assessment of growth characteristics, and the in vitro rhizome induction experiments. The statistical significance of the differences between the mean values was then assessed using Duncan’s multiple range test at *p* < 0.05. All statistical analyses were performed using SAS 9.4 software (SAS Institute Inc., Cary, NC, USA).

## 3. Results

### 3.1. Establishment of Culture

**Figure 1:**
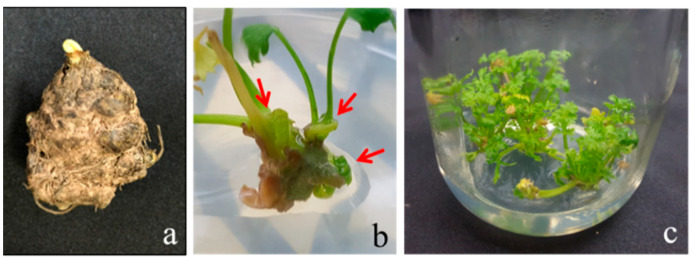
In vitro regeneration of *Cnidium officinale* plants on MS medium with 0.5 mg∙L^−1^ benzyl adenine (BA) and 30 g∙L^−1^ sucrose: (**a**) rhizome, (**b**) shoot development (arrows denote multiple shoots), and (**c**) plantlets.

**Figure 2:**
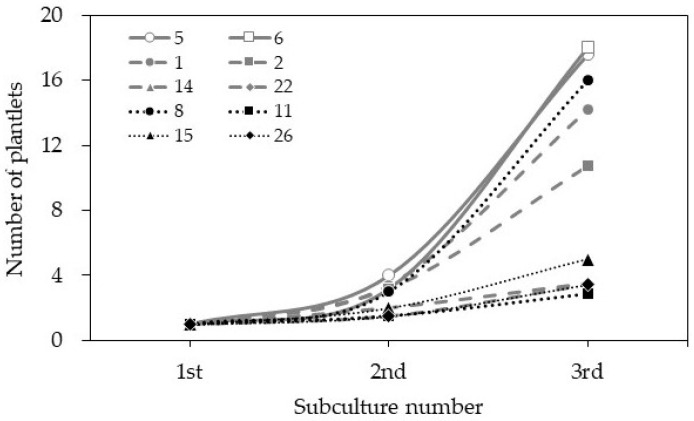
The number of plants regenerated from different clones of *Cnidium officinale* on MS medium with 5 mg∙L^−1^ benzyl adenine (BA) and 30 g∙L^−1^ sucrose over three 4-week passages of subculture.

The *Cnidium officinale* rhizome buds (Figure 1a) were cultured on MS medium, with 0.5 mg L^−1^ BA involved in swelling one week after inoculation, and produced multiple shoots in subsequent weeks (Figure 1b). The roots simultaneously formed at the basal shoots on the same medium, and plantlets with well-developed shoots and roots were obtained after 4 weeks of incubation (Figure 1c). The shoot proliferation rate varied from 1.5 to 3.4 times across the different clones. Among the ten clones used (clone nos. 1, 2, 5, 6, 8, 11, 14, 15, 22, and 25), clones 5 and 6 performed well and regenerated 16–18 shoots per explant after the third cycle (Figure 2).

### 3.2. Flow Cytometric Analysis of DNA Content in Different Clones of Cnidium officinale and Selection of Superior Clones with the Highest Regeneration Potential

**Figure 3:**
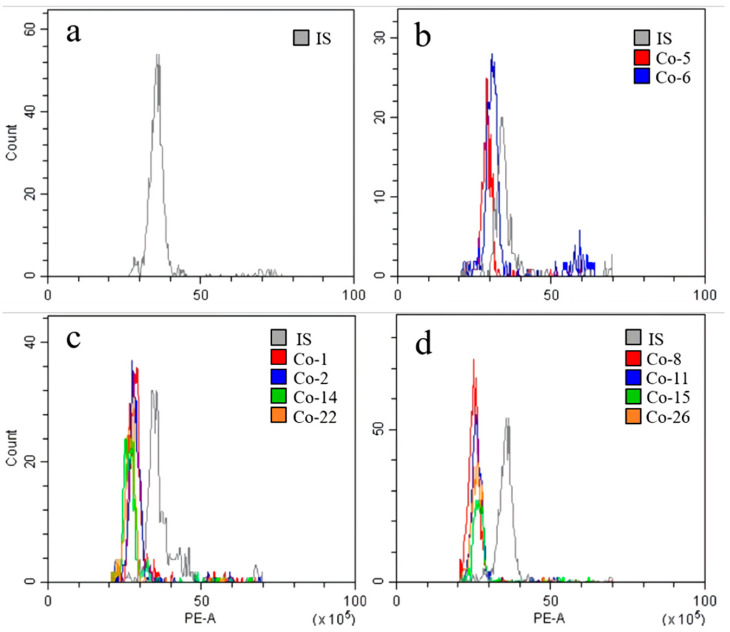
Analysis of 2C DNA content in different clones of *Cnidium officinale* plants using flow cytometry: histograms showing the DNA content of *Nicotiana tabacum* cv. xanthi for internal standard (IS) (**a**); clones with high DNA content (HD) (**b**); clones with moderate DNA content (MD) (**c**); and clones with low DNA content (LD) (**d**).

According to the chromosome count database (CCDB, http://ccdb.tau.ac.il/ (accessed on 19 September 2022) [26], the chromosome number of *Cnidium officinale* populations/clones (germplasm) is highly variable, and the chromosome numbers of 2n = 22, 33, 44 have been reported for this species. The chromosome count of the available *Cnidium officinale* germplasm/clones was not known; therefore, we carried out a flow cytometric analysis of the 2C DNA content of the different clones used in the current experiment and selected the superior clones for further regeneration experiments and utilization. The flow cytometric analysis data of the *Cnidium officinale* clones used in the current experiment are presented in Figure 3 and Table 1. The DNA values varied across the different clones, ranging from 7.68 to 8.60 pg/2C (Table 1). Based on such data, the clones were classified into clones with a high DNA content (HD, clones 5 and 6), clones with a moderate DNA content (MD, clones 1, 2, 14, and 22), clones with a low DNA content (LD, clones 8, 11, 15, and 26). The clones with HD, MD, and LD were compared based on their regeneration potential on MS medium with 0.5 mg L^−1^ BA by using rhizome bud cultures. The clones with a higher DNA content (clones 5 and 6) showed the highest regeneration potential in terms of the regeneration rate (Figure 2).

### 3.3. Comparison of Growth Characteristics of Different Clones

**Figure 4:**
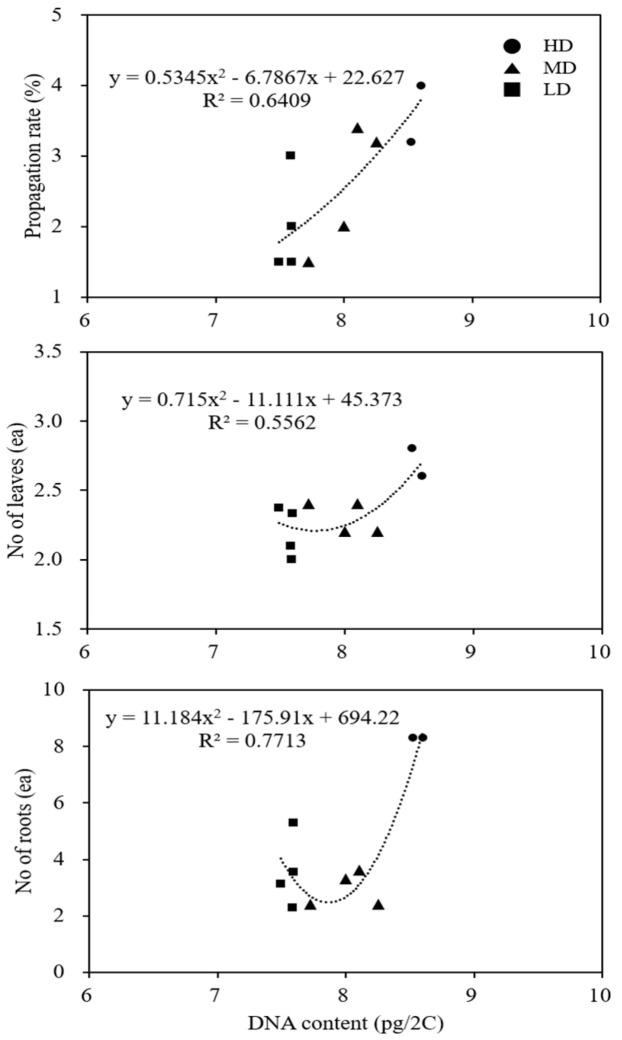
Correlation between DNA content and growth characteristics of ten clones of *Cnidium officinale*: HD, cones with high DNA content; MD, clones with moderate DNA content; and LD, clones with low DNA content.

The microshoots of several clones with known 2C DNA values were subcultured on MS medium with 0.5 mg L^−1^ BA and maintained for four weeks in culture to assess their growth characteristics and performance during in vitro propagation; the data are presented in Table 2. In terms of overall growth performance, i.e., fresh weight, the number of leaves, and the number of roots, the clones with higher DNA values performed well, and clone 5 was the best performer. A correlation analysis of the several clones with different DNA amounts with growth characteristics revealed that there was a positive correlation between the clones with the highest DNA content and propagation rate, the number of leaves, and the number of roots (Figure 4).

### 3.4. In Vitro Induction of Rhizome

**Figure 5:**
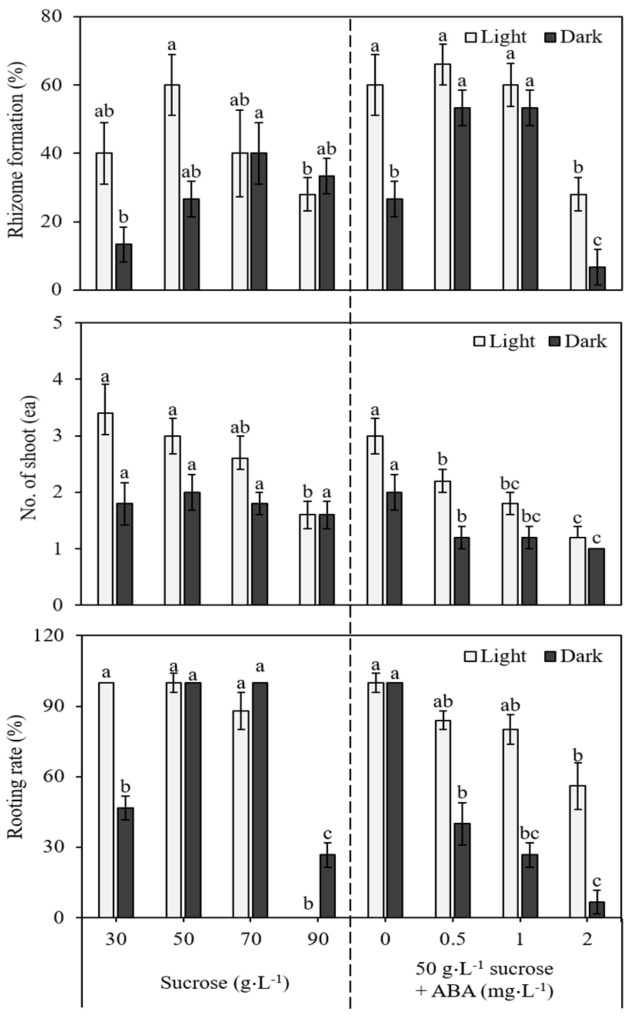
Effect of sucrose and ABA on rhizome induction of *Cnidium officinale* and under the same treatment (light vs. dark). Different letters in each bar significantly differ from each other as per DMRT (*p* < 0.05).

The results obtained for rhizome formation on media with different concentrations of sucrose and ABA, as well as for the light and dark incubation of the cultures, are presented in Figure 5. After two weeks of incubation, rhizome induction was evident at the base of the cultured shoots. Initially, the swelling of the shoot bases was observed, followed by the appearance of small rhizomes at the base within four weeks of incubation. Subsequently, the rhizomes were involved in the shoots’ growth, and roots simultaneously developed in the nodal regions of the rhizomes. Rhizome induction from the shoots was influenced by the levels of sucrose in the medium. The medium supplemented with 30 g L^−1^ to 50 g L^−1^ sucrose favored rhizome development (Figure 5). However, with a further increase in the concentration of sucrose from 50 g L^−1^ onwards (70 and 90 g L^−1^), there was a marked decrease in the percentage of rhizome formation and the rooting rate. Rhizomes formed on the medium in the presence of ABA, and the 0.5 mg L^−1^ and 1.0 mg L^−1^ ABA treatments were better in terms of the percentage of rhizome formation, the number of rhizomes forming shoots, and the rooting rate. A comparison of the light and dark treatments revealed that light incubation is stimulatory in inducing rhizomes from the shoots (Figure 5).

**Figure 6:**
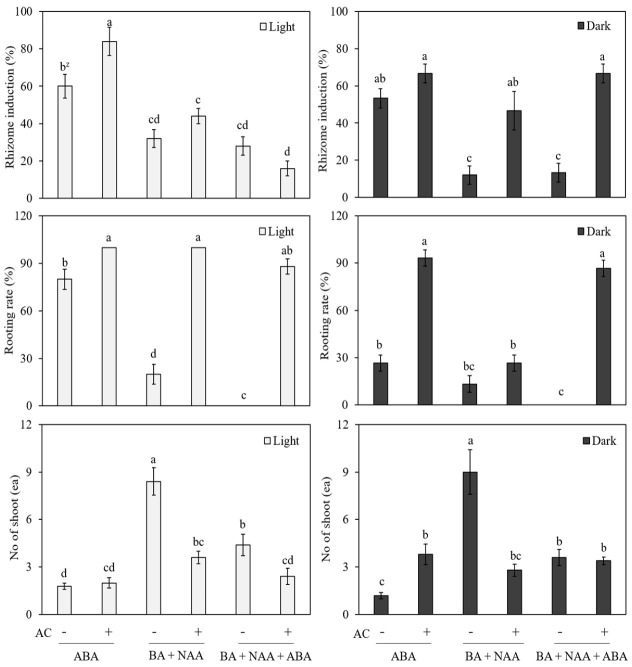
Effect of ABA, BA, and NAA with or without activated charcoal (AC) and under light or dark conditions on rhizome induction of *Cnidium officinale*. Different letters in each bar significantly differ from each as per DMRT (*p* < 0.05).

**Figure 7:**
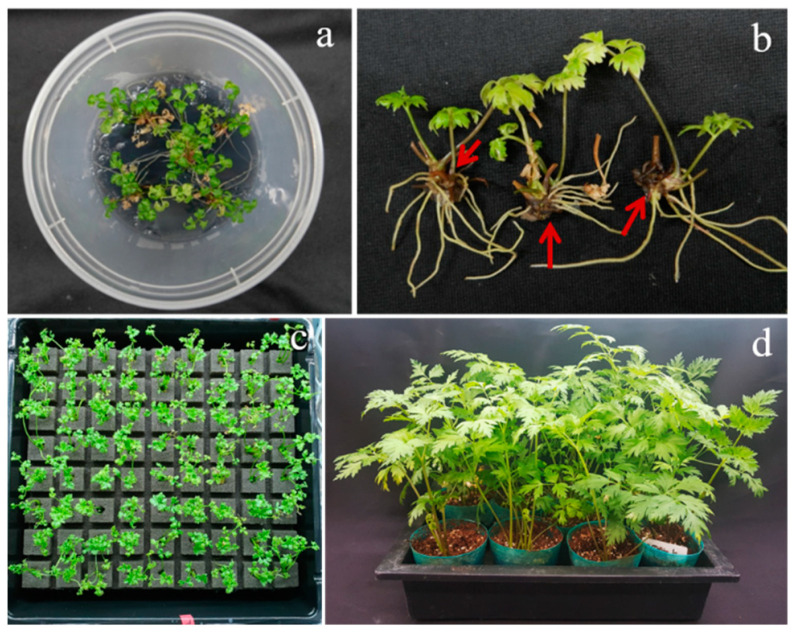
Rhizomes of *Cnidium officinale* developed with the in vitro-grown plantlets (**a**,**b**); (**c**) four-week-old plants growing with Growfoam; and (**d**) six-month-old plants growing in pots containing vermiculite.

In another set of experiments, we compared the effect of ABA (1.0 mg L^−1^) and the interaction of BA (1.0 mg L^−1^) + NAA (0.5 mg L^−1^) and BA (1.0 mg L^−1^) + NAA (0.5 mg L^−1^) + ABA (1.0 mg L^−1^) on rhizome induction for the in vitro-cultured shoots. At the same time, we also tested the effect of the light and dark treatments, along with hormonal combinations, with and without the addition of activated charcoal (1 g L^−1^), and the results are presented in Figure 6. Among these different treatments, the cultures that received light treatment performed better than those that received dark treatment, and again, the ABA treatment was far better than the BA + NAA and BA + NAA + ABA treatments. The results of the above experiments suggest that MS medium supplemented with 50 g L^−1^ sucrose, 1.0 mg L^−1^ ABA, and 1 g L^−1^ AC is good for the induction of rhizomes from the shoots of *Cnidium officinale* (Figure 7a,b).

### 3.5. Acclimatization

The in vitro-grown plants of *Cnidium officinale* after rhizome formation and rooting were acclimatized by planting them on Growfoam (Figure 7c), and the acclimatization rate of *Cnidium officinale* was 100%. After 6 months, the plants were transferred to plastic pots containing vermiculite, and, again, the survival rate was 100% (Figure 7d).

## 4. Discussion

*Cnidium officinale* is an important medicinal herb that is utilized in the traditional medicinal systems of China, Japan, and Korea. This plant is propagated by splitting rhizomes since it does not set seeds in nature; however, this method of propagation is responsible for the transmission of viral particles, which results in a loss of the yield and the quality of the rhizomes harvested. In the current study, we developed a successful plant regeneration method by using rhizome buds and by employing an MS medium containing 0.5 mg∙L^−1^ BA. Multiple shoots regenerated from the rhizome buds and were involved in rooting within four weeks of culture. This simple method was useful in regenerating plants, resulting in several clones of *Cnidium officinale*, which were maintained in a germplasm repository. The shoot propagation rate varied from 1.5 to 3.4 times across the different clones. Among the ten clones used (clone nos. 1, 2, 5, 6, 8, 11, 14, 15, 22, and 25), clones 5 and 6 performed well and regenerated 16–18 shoots per explant after the third cycle. Lee et al. [1] reported induction somatic embryogenesis in *Cnidium officinale* from immature flower cultures on MS medium supplemented with 1.0 mg L^−1^ 2,4-D and 0.1 mg L^−1^ BAP. The embryos that developed were converted into plantlets on MS medium containing 0.1 mg L^−1^ BAP and 3.0 mg L^−1^ BAP GA_3_. Similarly, Adil et al. [27] described recurrent somatic embryogenesis in *Cnidium officinale* on MS medium containing 1.5 mg L^−1^ 2,4-D and 0.5 mg L^−1^ BAP. The above results suggest that the induction of both organogenesis and somatic embryogenesis is feasible for the micropropagation of *Cnidium officinale.*

The flow cytometric estimation of nuclear DNA content is a useful tool in the assessment of medicinal plants regenerated in vitro [28]. In the current study, the clones containing a high amount of DNA, i.e., clones 5 and 6, showed better growth characteristics in terms of fresh weight, the number of leaves, and the number of roots; they were also better performers in terms of the propagation rate. DNA content variations and associated divergence in biological characteristics have also been reported in the natural populations and field-grown plants of *Erianthus arundinaceus* [29] and *Rauvolfia sepentina* [30].

The induction of rhizomes during in vitro propagation is an ideal system that helps in the early establishment of plants in fields. An in vitro-induced rhizome produces both shoots and roots on germination, giving rise to a complete plantlet and, thus, acting as a seed for the large-scale propagation of rhizomatous plants [24]. Plant growth regulators, sucrose, and light and dark conditions are known to affect the in vitro induction of rhizomes in many rhizomatous plants, including *Curcuma longa* [23], *Bambusa bambos* var. *gigantea* [24], *Kaempferia galanga*, and *K. rotunda* [22].

The results of the present experiments revealed that the supplementation of medium with a suitable sucrose concentration (50 g L^−1^) is suitable for rhizome induction in *Cnidium officinale.* Similar to the current results, 50 g L^−1^ has been found to be suitable for the induction of rhizomes from the in vitro-raised shoots of *Bambosa bambos* var. *gigantea* [24]. Sucrose acts as a carbon source and as an osmotic agent, and since storage organs are abundant in carbohydrates, the appropriate concentration of sucrose is essential for the induction and growth of rhizomes in vitro [31,32]. The supplementation of ABA (0.5 and 1.0 mg L^−1^) favored the induction and development of rhizomes from the microshoots of *Cnidium officinale*; however, an increased concentration of ABA (2.0 mg L^−1^) was not beneficial for the induction and growth of rhizomes. Similar to the current observations, Yang et al. [33] reported the promotive effect of ABA on rhizome induction in *Nelumbo nucifera*. In the current study, light favored rhizome induction from microshoots, whereas the negative effect of the dark treatment was evident. The effect of light on rhizome formation observed in the present study is comparable to that observed in the study conducted by Sakamura et al. [34], where the requirement of continuous light during rhizome formation in *Zingiber officinale* was reported. The influence of the combined effect of BA, NAA, and ABA did not favor the formation of rhizomes from the microshoots of *Cnidium officinale*. In contrast to the current results, BA and NAA have been shown to influence the process of in vitro rhizome formation in *Cymbidium goeringii* [35].

The plantlets with well-developed rhizomes and roots showed 100% survival upon transplantation to Growfoam and subsequently to vermiculite. The protocol for the in vitro induction of multiple shoots and subsequent rhizome formation in *Cnidium officinale* can be utilized by commercial growers for the production of disease-free clones on a large scale.

## Figures and Tables

**Table 1:** DNA content of ten in vitro-grown clones of *Cnidium officinale*.

Group	Clone	Median	CV	DNA Content ^z^ (pg/2C)
HD ^y^	5	2,988,019.06	5.9%	8.60 ± 0.15a ^x^
HD	6	2,961,047.90	6.0%	8.52 ± 0.20ab
MD	1	2,816,142.06	5.8%	8.10 ± 0.10abc
MD	2	2,869,026.08	5.8%	8.26 ± 0.41abc
MD	14	2,779,891.16	6.0%	8.00 ± 0.24abc
MD	22	2,683,071.82	6.8%	7.72 ± 0.28bc
LD	8	2,634,563.66	6.8%	7.58 ± 0.23c
LD	11	2,602,247.30	6.3%	7.49 ± 0.20c
LD	15	2,638,274.26	6.7%	7.59 ± 0.38c
LD	26	2,636,395.84	6.1%	7.68 ± 0.18c

^z^ Internal standard for calculating DNA content is *Nicotiana tabacum* cv. xanthi (10.07 pg/2C). ^y^ HD: high DNA content, MD: moderate DNA content, LD: low DNA content. ^x^ Different letters indicate significantly different values at *p* < 0.05 according to Duncan’s multiple range test (*n* = 5).

**Table 2:** Growth characteristics of ten in vitro-grown clones of *Cnidium officinale*.

Clone	Plant Height (mm)	Fresh Weight (mg/Plantlet)	No. of Leaves (ea/Plantlet)	No. of Roots (ea/Plantlet)	Root Length (mm)
1	24.5 ± 0.9bc ^z^	168.0 ± 12.1ab	2.4 ± 0.3	3.6 ± 0.4bc	11.1 ± 1.3a
2	18.9 ± 0.7ef	160.0 ± 18.5ab	2.2 ± 0.2	2.4 ± 0.3c	4.6 ± 0.3d
5	21.4 ± 1.2cde	230.0 ± 23.3a	2.6 ± 0.5	8.3 ± 0.3a	6.4 ± 0.9cd
6	22.1 ± 1.0bcde	198.0 ± 18.6ab	2.8 ± 0.2	8.3 ± 1.0a	10.3 ± 0.6ab
8	19.9 ± 0.9def	206.0 ± 14.6a	2.1 ± 0.2	2.3 ± 0.6c	5.9 ± 1.7cd
11	17.6 ± 0.8f	202.0 ± 13.4a	2.4 ± 0.2	3.1 ± 0.9bc	6.3 ± 0.5cd
14	25.3 ± 1.6ab	204.0 ± 23.4a	2.2 ± 0.3	3.3 ± 0.5bc	7.2 ± 1.3cd
15	20.8 ± 1.2def	210.0 ± 29.4a	2.3 ± 0.2	3.6 ± 0.5bc	8.0 ± 0.7bc
22	27.9 ± 1.2a	208.0 ± 15.3a	2.4 ± 0.3	2.4 ± 0.3c	8.2 ± 1.0abc
26	22.4 ± 1.3bcd	134.0 ± 15.1b	2.0 ± 0.1	5.3 ± 0.8b	7.9 ± 1.2bc

^z^ Different letters indicate significantly different values at *p* < 0.05 according to Duncan’s multiple range test (*n* = 5).

## Data Availability

Not applicable.
